# Endovascular balloon-assisted liquid embolisation of soft tissue vascular malformations: technical feasibility and safety

**DOI:** 10.1186/s42155-021-00236-4

**Published:** 2021-06-08

**Authors:** Anthony Lamanna, Julian Maingard, Grace Florescu, Hong Kuan Kok, Dinesh Ranatunga, Christen Barras, Michael J. Lee, Duncan Mark Brooks, Ashu Jhamb, Ronil V. Chandra, Hamed Asadi

**Affiliations:** 1 Interventional Radiology Service, Department of Radiology, Austin Hospital, Melbourne, Australia; 2 Interventional Neuroradiology Service, Department of Radiology, Austin Hospital, Melbourne, Australia; 3 Interventional Neuroradiology Unit, Monash Imaging, Monash Health, Melbourne, Australia; 4 School of Medicine, Faculty of Health, Deakin University, Waurn Ponds, Australia; 5 Interventional Radiology Service, Northern Health Radiology, Melbourne, Australia; 6 South Australian Health and Medical Research Institute, Adelaide, Australia; 7 Department of Radiology, Beaumont Hospital and Royal College of Surgeons in Ireland, Dublin, Ireland; 8 Stroke Division, Florey Institute of Neuroscience and Mental Health, University of Melbourne, Melbourne, Australia; 9 Interventional Neuroradiology Service, Department of Radiology, St Vincent’s Hospital, Melbourne, Australia

**Keywords:** Arteriovenous malformation, Embolisation, Liquid embolisation, Soft tissue, Onyx, PHIL

## Abstract

**Purpose:**

Arteriovenous malformations (AVMs) are abnormal communications between arteries and veins without an intervening capillary system. The best endovascular treatment option for these is unclear and may involve multiple staged procedures using a variety of embolic materials. We report our initial experience using a modified version of a previously published neurointerventional technique to treat soft tissue AVMs with single-stage curative intent.

**Materials and methods:**

Soft tissue AVMs treated endovascularly using either sole arterial or combined arterial and venous balloon-assisted techniques with liquid embolic agents were retrospectively identified over a 3.5 year period (January 2017 to June 2020)) at two centres. Clinical, pre-operative radiological, procedural technical and post treatment details were recorded.

**Results:**

Seven patients were treated for symptomatic soft tissue arteriovenous malformations. These AVMs were located in the peripheral limbs (five), tongue (one) and uterus (one). Curative treatment was achieved in 6/7 patients with one patient requiring a second treatment approximately 1 year later. A variety of liquid embolisation agents (LEAs) including sclerosants and polymers were used. Clinical success rate was 100% following treatment. One patient experienced expected temporary post-operative tongue swelling requiring tracheostomy occurred following embolisation of the lingual AVM. A minor complication in a second patient was due to an access site haematoma developed following treatment of the hand AVM requiring surgical intervention. No long-term sequelae or additional complications were observed.

**Conclusion:**

Endovascular arterial and venous balloon assisted LEA embolization of soft tissue AVMs with curative intent is feasible. This technique may provide an alternative treatment option for achieving durable occlusion for complex soft tissue AVMs.

## Introduction

Vascular malformations (VMs) are abnormal networks of dysplastic vessels that may involve arteries, veins, capillaries or lymphatics. Peripheral vascular malformations occur in the extremities, usually within the skin and subcutaneous tissues. Several classification systems that define VMs have been published, the first by Mulliken and Glowacki in 1982 (Mulliken & Glowacki, 1982a; Gilbert et al., 2017; Mulligan et al., 2014; Ernemann et al., 2010). The International Society for the Study of Vascular Anomalies (ISSVA) classification is currently one of the most used classification systems whereby vascular anomalies are divided into vascular tumours and VMs (Dasgupta & Fishman, 2014; Mulliken & Glowacki, 1982b). VMs are further divided into simple malformations and combined malformations depending on which types of vessels are involved. Arteriovenous malformations (AVMs) are VMs involving arteries and veins that communicate via a central nidus in the absence of normal capillaries (Lee et al., 2013). Venous malformations are comprised of an ectatic network of dysplastic veins. Haemodynamic flow through a VM is also used to classify these into ‘high-flow’ and ‘low-flow’. AVMs are typically high-flow due to the high pressures within the feeding arterial vessels while low-flow malformations include venous malformations along with numerous other VMs (Jackson et al., 1993).

Endothelial cells contribute to vascular remodelling and are thus important therapeutic targets of AVM treatment, in particular at the venous sump which recruits collateral flow (Gilbert et al., 2017; Mulliken & Glowacki, 1982b). Interventional techniques aim to deliver therapeutic agents whilst targeting the venous sump and avoiding damage to adjacent tissue (Legiehn & Heran, 2006). The rate of injection, flow rate in the vascular bed, size of treated area, dwell time of the agent and its concentration are important factors in determining adequate endothelial damage. The complex configuration of AVMs often poses a technical challenge when attempting to target the venous sump (Rosen et al., 2013). Nidal access is achieved via a transarterial, transvenous or direct puncture approach, with subsequent infiltration of liquid embolic agent (LEA) (Conway et al., 2015).

AVMs were previously managed with open surgical ligation of arterial and venous outflow vessels. Early experiences demonstrated that intuitive ligation of feeding vessels worsened the condition, leading to recruitment of collateral vessels and increased vascularity secondary to local ischaemic effects (Lee et al., 2013). Currently, endovascular embolisation is the mainstay of treatment. This is typically performed with dimethyl sulphoxide (DSMO) LEAs such as Onyx (Medtronic, Minneapolis, MN, USA), an ethylene vinyl alcohol copolymer (EVOH), or PHIL (Microvention, Aliso Viejo, CA, USA), a precipitating hydrophobic injectable LEA. Poly-vinyl alcohol (PVA) particles and acrylic glue such as Glubran (GEM Srl, Viareggio, Italy) may also be used. Coils and plugs are rarely used as they prevent access if further treatment is warranted (Madani et al., 2015).

Further to the choice of embolic, the type of delivery device and technique impacts the ability of the interventionalist to achieve successful treatment. The need for flow control in AVM treatment is essential to achieve stasis within the nidus and allow the embolic agent to be deposited in the desired position. This can been addressed using a venous tourniquet or outflow embolisation prior to nidus embolisation (pressure cooker and modified pressure cooker techniques) (Chapot et al., 2014; Zhang et al., 2017; Abud et al., 2016). An occlusion balloon may also be employed, although not routinely available in all catheter sets (Arslan, 2000; Gentric et al., 2015; Kim et al., 2017; Vollherbst et al., 2018a; Hyodoh et al., 2005). Larger catheters without balloon functions present reported issues with embolic reflux into non-target vessels, vascular perforation, adhesion of catheter to vessels and need for multiple procedures to achieve resolution (Gungor et al., 2017; Weber et al., 2007).

Dual-lumen balloon catheters are useful in achieving targeted delivery of embolic agents by occluding either the feeding artery alone (‘balloon-assisted pressure cooker technique’) or in conjunction with the draining vein (‘double balloon-assisted pressure cooker technique’). Scepter C/XC (Microvention, Aliso Viejo, CA, USA), Occlusafe (Terumo, Tokyo, Japan), Ascent (DePuy Synthes, Raynham, MA, USA) and Eclipse 2 L (Balt Extrusion, Montmorency, France) are examples of these types of devices. The use of Scepter devices with PHIL and Onyx embolic agents has been validated in intracranial AVMs but poorly explored in peripheral VMs (Trivelato et al., 2018; Jagadeesan et al., 2013). The aim of this study is to evaluate balloon-assisted pressure cooker techniques in the treatment of peripheral soft tissue AVMs at two centres.

## Materials and methods

A retrospective study of treated peripheral soft tissue VMs over a 3.5 year period (January 2017 to June 2020) was conducted. Patients were included if they were endovascularly treated for a peripheral VM using LEAs within the study period. Patient demographics, procedure indications, technical procedural details, complications, baseline and follow-up indices were reviewed from the hospital radiology and patient information systems and were recorded up to most recent follow up. AVMs treated using either sole endovascular arterial balloon assisted or combined endovascular arterial and venous balloon assisted embolisation techniques were included in the study. Specific clinical and technical details for each case are described below.

## Result

### Case 1 – lower limb low-flow venous malformation

**Fig. 1:**
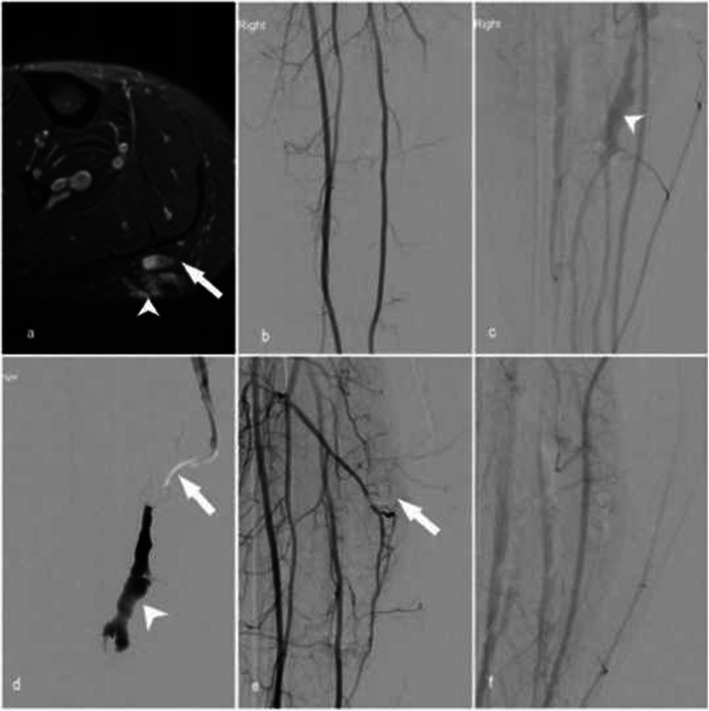
Patient 1. a) Axial post contrast T2 weighted fat saturated MRI demonstrating a lobulated slow-flow vascular malformation in the posterior calf measuring 5.7 cm with fluid haematocrit levels (arrowhead) with dilated draining veins (arrow). b) Digital subtraction angiography did not reveal arterial inflow compatible with a slow-flow venous malformation. c) Venous phase of the angiogram showing venous pouch d) Digital subtraction venography showing retrograde venous access with 6Fr intermediate catheter (Sofia, Microvention, etc) (arrow) placed within the venous pouch (arrowhead). e) Arterial inflow was then controlled using a pressure cooker technique with inflation of a dual balloon catheter (Scepter C, Microvention, etc) (arrow). 6 ml sodium tetradecyl sulphate 3% was injected from the venous side with f) subsequent arteriogram confirming no evidence of arterial leak or distal embolization and successful occlusion of the venous malformation

The patient presented with a long-standing right calf vascular malformation and was referred for treatment due to recent thrombosis and increased pain particularly with prolonged standing. Multi sequence magnetic resonance (MR) imaging revealed a lobulated vascular malformation in the posterior calf measuring 5.7 cm with fluid haematocrit levels and dilated draining veins. No convincing arterial feeders and nidus were demonstrated, consistent with a small low-flow venous malformation (Fig. 1).

Under general anaesthesia (GA), a right antegrade common femoral artery (CFA) puncture was performed followed by placement of a 6Fr introducer sheath (Terumo, Tokyo, Japan). Superselective digital subtraction angiography (DSA) of the tibioperoneal trunk was performed via a 6Fr intermediate guiding catheter (Sofia, Microvention). Following a retrograde common femoral vein (CFV) puncture, venography was performed using a 6Fr intermediate catheter (Sofia, Microvention) revealing a superficial venous anomaly, with multiple small and large venous pouches and patent deep venous drainage. No arterial feeders or nidus were demonstrated on DSA. A dual-lumen microballoon occlusion catheter (Scepter C, Microvention) was used to control arterial inflow using a balloon-assisted pressure cooker technique. A total of 6 ml 3% sodium tetradecyl sulphate was injected with contrast under fluoroscopic via the balloon catheter with subsequent balloon DSA confirming no evidence of arterial leak, non-target embolisation or significant filling of the venous lakes. The patient was discharged the following day with no complications. At 3 month follow-up, there was complete symptomatic resolution with no residual vascularity present on corresponding MRI.

### Case 2 – lower limb high-flow arteriovenous malformation

**Fig. 2:**
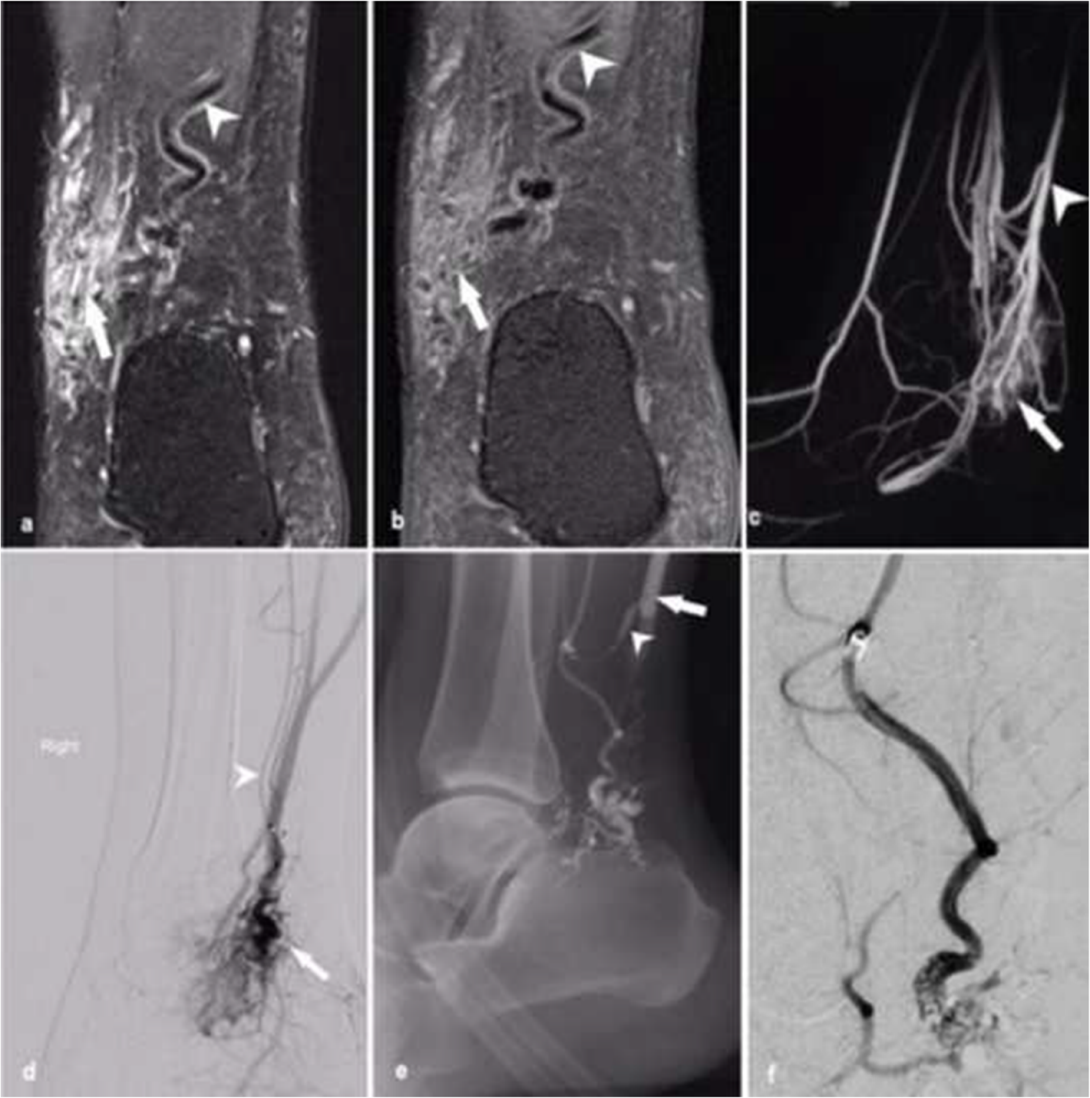
Patient 2. a) Coronal fat saturated T2, b) post-contrast fat saturated T1-weighted MRI and c) time resolved MRA image of the right foot showing the AVM including nidus (arrow) and early drainage into adjacent posterior tibial veins (arrowhead). d) DSA via a 5Fr catheter (Sofia, Microvention, Aliso Viejo, California) in the arterial feeder arising from the distal posterior tibial artery above the ankle (arrowhead) demonstrating the nidus (arrow). e) the balloon-occlusion catheter (Scepter XC, Microvention Inc., Aliso Viejo, CA, USA) can be appreciated to lie within the feeding artery (arrowhead) with venous outflow controlled with a 10 × 80 mm Copernic super compliant balloon (arrow) and f) post-embolisation angiogram demonstrated nidus staining with absence of residual nidus

The patient presented with a longstanding right ankle AVM which had become increasingly painful. Postcontrast fat-saturated MR angiography demonstrated a high-flow AVM with a nidus along the medial aspect of the ankle, with reconstructed 4-dimensional maximal intensity projections showing multiple arterial feeders and venous outflow channels (Fig. 2).

Under GA, antegrade CFA and retrograde CFV access was achieved using 6Fr introducer sheath (Terumo). DSA confirmed the MR angiography findings of a large nidus with posterior tibial arterial feeders and multiple deep venous posterior tibial vein outflow channels. A 6Fr intermediate catheter (Envoy DA, Codman Neuro, Johnson&Johnson Medical Ltd., Workingham, United Kingdom) and 5Fr intermediate catheter (Sofia, Microvention) was advanced into the distal posterior tibial artery followed by a dual-lumen microballoon occlusion catheter (Scepter XC, Microvention) into the main arterial feeder. Following this, from the venous end, an intermediate catheter and a 10 × 80 mm super compliant balloon catheter (Copernic RC, BALT Extrusion, Montmonrency, France) were used to access and occlude the main draining vein. Arterial inflow and venous outflow to-from the nidus were controlled using a double balloon-assisted pressure cooker technique so that the nidus could be isolated for deep nidal penetration by embolic material. High-pressure embolisation was performed from the arterial side using PHIL until the nidus was completely penetrated. The venous occlusion balloon remained inflated for several minutes, however at the conclusion of the procedure some minor superficial venous penetration was observed without any other deep venous compromise. There was no evidence of non-target arterial embolisation or reflux. The patient was discharged the following day with no complication. Symptoms were substantially improved at 6-month clinical review with only mild symptoms after ambulating 2 km. Follow-up MR angiography at 6 months revealed a reduction in size of the AVM with some minor residual arterial feeders without significant shunting. Given improved symptomatology no further treatment was performed.

### Case 3 – upper limb high-flow arteriovenous malformation

**Fig. 3:**
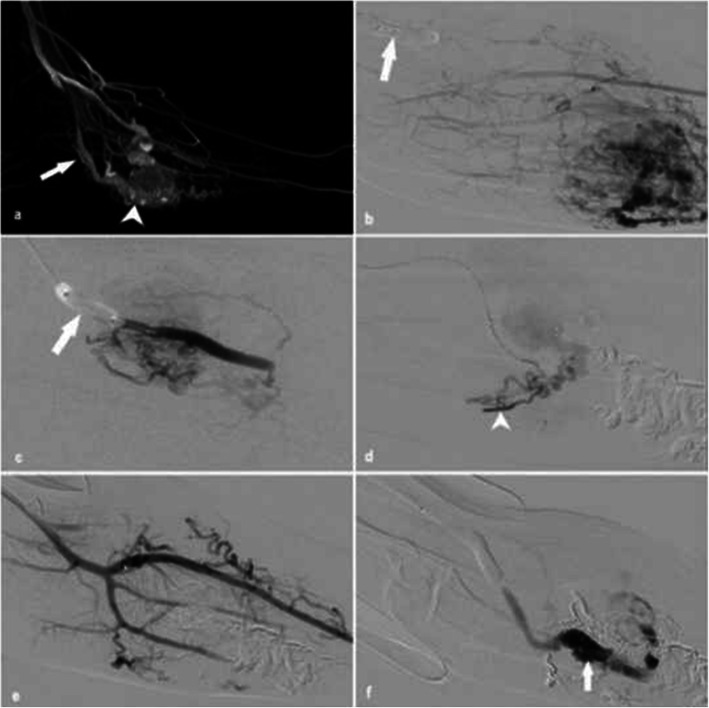
Patient 3. a) Time resolved MRA of the right elbow demonstrates arterial feeder with a nidal component of the AVM (arrowhead) and large venous varicosities (arrow). b) Digital subtraction angiogram at the time of arterial injection demonstrates a large high flow nidus arising from the radial artery. Note that simultaneous venous access has been established (arrow) c) Arterial inflow occluded with the (Scepter C, Microvention Inc., Aliso Viejo, CA, USA) occlusion balloon (arrow). d) angiography post initial treatment demonstrated residual nidus (arrow) and further treatment using Onyx and PHIL was performed. e) and f) arterial and venous injections respectively with remaining venous flow demonstrated (arrow). 3% sodium tetradecyl sulphate with contrast was subsequently injected to occlude venous outflow

A patient who presented with right elbow pain and swelling was found to have a high-flow AVM on MR imaging, with a complex nidus and at least two large draining varicose veins overlying the extensor compartment of the forearm (Fig. 3).

**Fig. 4:**
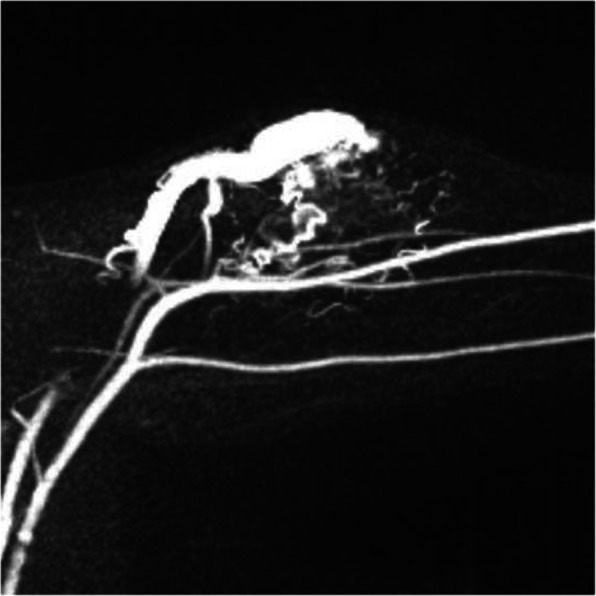
Time resolved MRA performed at 3 months postprocedure demonstrating decrease in arterial inflow channels and in size of nidal component. This patient had clinical recurrence of the AVM at 2 years requiring retreatment

Under GA, right-sided retrograde CFA and CFV punctures were performed with placement of 8Fr introducer sheaths (Terumo). An 8Fr multi-lumen access catheter (Arrow, Teleflex Medical, Gurnee, Illinois) was placed in the right brachial artery and 6Fr intermediate catheter (Sofia-Plus, Microvention Inc.) were advanced toward the AVM nidus from both venous and arterial sides. Venous outflow was controlled with a 10 × 80 mm super compliant balloon catheter (Copernic RC, BALT Extrusion) catheter in the mid right brachial vein in addition to manual compression using a blood pressure cuff inflated to 90 mmHg. A dual-lumen microballoon occlusion catheter (Scepter XC, Microvention) was used to localise and compartmentalise several separate compartments within the AVM. Embolisation of the AVM nidus was performed using Onyx and PHIL embolic agents to good effect using a double balloon-assisted pressure cooker technique. Non-target embolisation was not observed. A postprocedural venogram demonstrated ongoing opacification of peripheral varicosities. These were obliterated under fluoroscopic guidance from the venous side using 3% sodium tetradecyl sulphate with contrast which was injected through a jailed microcatheter (SL10, Stryker Neurovascular, Fremont, CA, USA) from the venous side, again with controlled venous outflow. At clinical follow up the patient had symptomatically improved and had normal distal perfusion. Three month MRI demonstrated reduced shunting with only small residual arterial feeders (Fig. 4).

Two years following treatment, there had been clinical recurrence of the AVM and repeat endovascular treatment was performed. Again under GA, right antegrade CFV and retrograde CFA access using 10Fr and 8Fr access sheaths (Terumo) were achieved. DSA performed through a 5Fr diagnostic catheter placed in the right brachial artery revealed recurrence of the AVM with multiple arterial feeders originating from the ulnar artery draining into a solitary dilated vein. The diagnostic catheter was exchanged for an 8Fr multi-lumen access catheter (Arrow, Teleflex Medical) and a 6Fr intermediate catheter (Sofia-Plus, Microvention Inc.) was placed in the ulnar artery. The dilated draining vein was punctured directly and accessed via Seldinger technique using a 6Fr sheath and a 6Fr intermediate catheter (Sofia-Plus, Microvention Inc.) was advanced towards the nidus from the venous side. Manual compression and balloon occlusion of the outflow vein was performed using a 10 × 80 mm super compliant balloon catheter (Copernic RC, BALT Extrusion) while arterial inflow was controlled using a dual-lumen microballoon occlusion catheter (Scepter XC, Microvention; double balloon-assisted pressure cooker technique). With the nidus isolated, liquid embolisation using Onyx and PHIL embolisation was performed. Complete occlusion was achieved with no immediate complications. At 3-month follow up, the patient reported no ongoing symptoms and there was no clinical evidence to suggest AVM recurrence. A repeat MR is planned for 6 months post-treatment.

### Case 4 – lingual venous malformation

A patient presented with recurrent oral bleeding and airway symptoms. Multi-sequence MRI revealed a large vascular malformation with predominant arterial supply arising from the lingual branch of the common carotid artery.

**Fig. 5:**
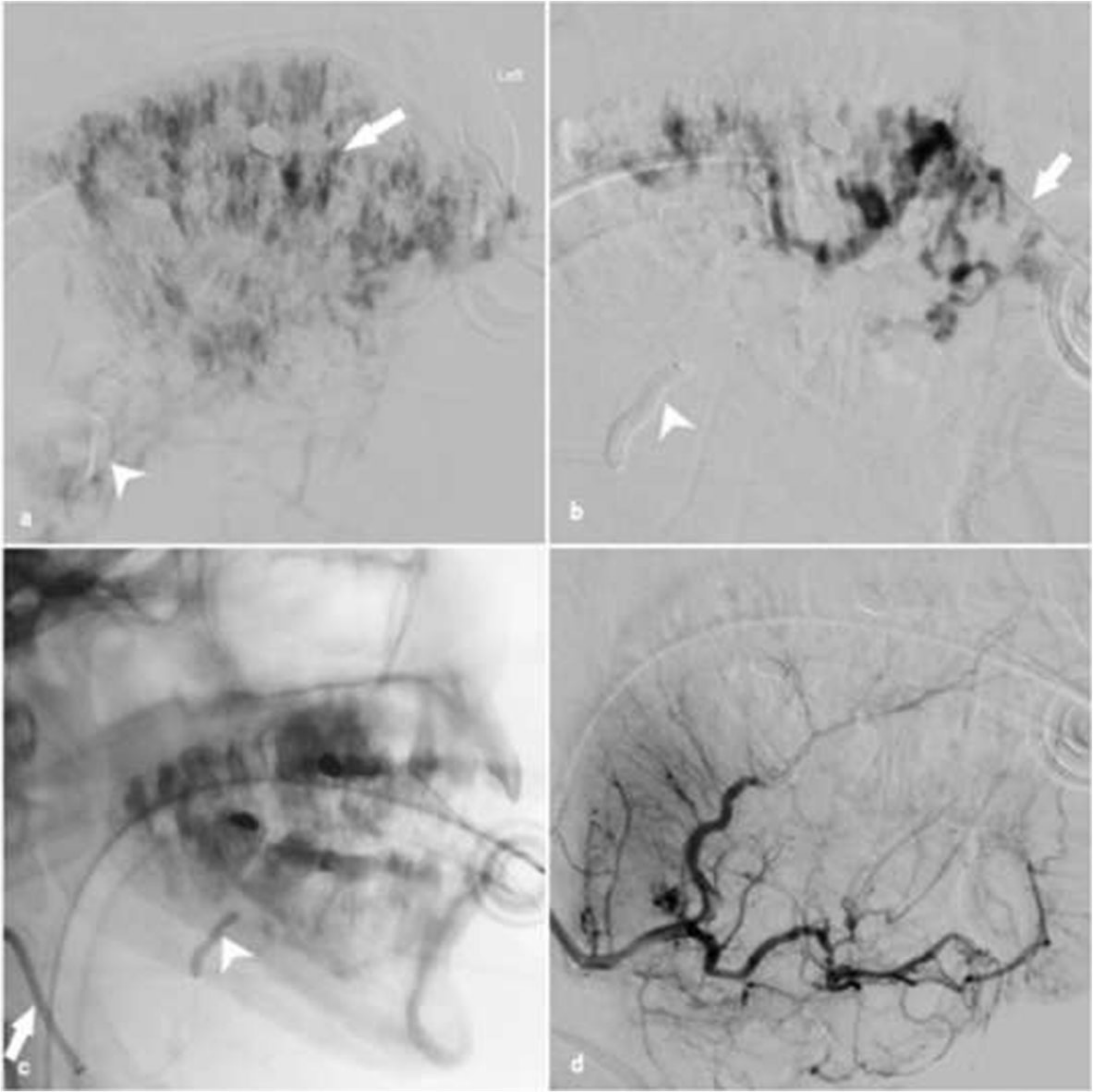
Patient 4 (tongue AVM). A) Arterial injection of the lingual artery into Envoy DA and Scepter C (arrowhead) demonstrating prominent parenchymal blush over the dorsum of the tongue (arrow) with no distinct nidus, most in keeping with a predominant low flow venous malformation. b) Pressure cooker technique with occlusion of arterial inflow using the Scepter C balloon catheter (arrowhead) with percutaneous access using a Chiba needle (arrow) and injection of 4 ml 3% liquid sodium tetradecyl sulphate. C) Unsubtracted image demonstrating embolic material in situ with stasis of flow. Envoy DA catheter (arrow) and Scepter C (arrowhead) are also visible d) Final check angiogram shows no evidence of residual venous malformation

Given significant swelling, the patient was intubated and the procedure was performed under GA. The patient was prescribed 4 mg daily of dexamethasone pre-procedure and was given a stat dose of 8 mg IV dexamethasone at the commencement of the procedure. Right-sided CFA and CFV access was achieved followed by insertion of 8Fr introducer sheaths (Terumo). DSA revealed a prominent parenchymal blush over the dorsum of the tongue with no distinct nidus, suggestive of a predominant low-flow venous VM (Fig. 5). Significant venous pooling and varicosities were noted. A 6Fr intermediate catheter (Sofia-Plus, Microvention Inc.) was placed in the middle of the lingual artery. A dual-lumen microballoon occlusion catheter (Scepter C, Microvention) was then inflated into a distal branch of the lingual artery to compartmentalise the malformation using a balloon-assisted pressure cooker technique, leading to flow arrest and delayed venous clearance. Percutaneous access was then performed with a 22 gauge Chiba needle and 4 ml 3% sodium tetradecyl sulphate was injected into the malformation. The patient was transferred to the intensive care unit (ICU) for expected airway swelling where extubation failed, requiring tracheostomy formation. The patient was treated with high-dose IV dexamethasone (8 mg BD). As swelling subsided, the patient was extubated and eventually discharged from hospital with no complications. Follow up MRI demonstrated a significant reduction in size of the lingual compartment of the AVM with residual arterial flow and patency of the floor of mouth component, albeit smaller compared to preprocedural imaging. The patient remains mildly symptomatic with fullness at the floor of the mouth and occasional ulceration and bleeding. Repeat 6 month imaging and repeat clinical review have been planned given the expected risks of repeat airway swelling with repeated embolisation.

### Case 5 – hand high-flow arteriovenous malformation

**Fig. 6:**
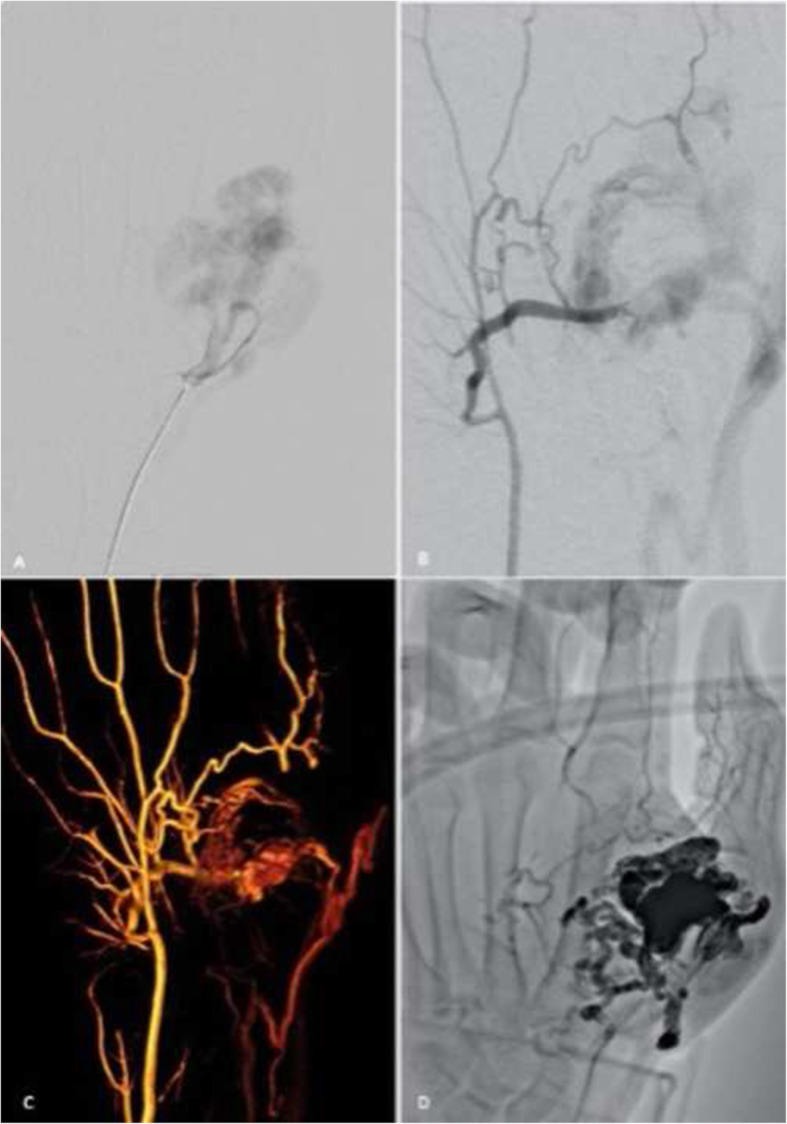
Patient 5 (wrist AVM). a and b) Preprocedural digital subtraction angiography demonstrated a large, multi-compartmental, complex nidal and fistulous AVM with extensive deep and superficial palmar arch feeders and multiple venous outflow vessels. 4D reconstruction of the AVM are shown in c. Liquid embolisation using Onyx and PHIL shown in d

A patient presented with right arm pain and marked visible collateral veins secondary to a large high-flow vascular malformation of the right hand. Non-contrast and angiographic CT images revealed a large AVM with the predominant feeding arterial supply arising from the ulnar artery with multiple draining veins including the basilic, cephalic and brachial veins. Preprocedural DSA was also performed for treatment planning, confirming a large, multi-compartmental, complex nidal and fistulous AVM with extensive deep and superficial palmar arch feeders and multiple venous outflow vessels (Fig. 6).

The procedure was performed under GA. Retrograde and antegrade access of the right CFA and CFV respectively using 8Fr Terumo access sheaths were achieved using the Seldinger technique, followed by retrograde access of the right brachial vein. Achievement of venous access was extremely difficult with multiple inadvertent arterial cannulations. Once access was obtained, an 8Fr multi-lumen access catheter (Arrow, Teleflex Medical) was placed in the ulnar artery. 6Fr intermediate catheters (Sofia-Plus, Microvention Inc.) were advanced toward the AVM from both venous and arterial sides and extensive embolisation of the nidus was performed with Onyx and PHIL using a double balloon-assisted pressure cooker technique. Completion angiography was suggestive of thromboembolism in the 1st and 2nd intermetacarpal arteries, demonstrated by slow flow. Aspiration thrombectomy using a 5Fr catheter (Sofia, Microvention Inc.) was performed and slow intraarterial infusion of urokinase (600,000 units) and tirofiban (8 mg) was administered. A total of 10,000 units of IV and IA heparin was also given. Despite reversal of heparin and prolonged manual compression of the brachial access site, significant arm swelling occurred. A postprocedural CT angiogram demonstrated an anterior forearm haematoma, brachial artery dissection and pseudoaneurysm with active bleeding. A small AVM residium was also present. The patient underwent surgical evacuation of the haematoma and repair of a venotomy and arteriotomy. No surgical complications occurred. At 6 month follow-up, the patient had returned to baseline with respect to hand function with no clinical AVM recurrence.

### Case 6 – uterine arteriovenous malformation

A patient presented with 3 weeks of pervaginal (PV) bleeding on a background of a termination of pregnancy with dilation and curettage 11 months prior. Transabdominal and transvaginal pelvic ultrasounds were performed which demonstrated a hypervascular region of myometrium in the uterine fundus measuring 1.5 cm in diameter, extending down to the endometrium. Multisequence and multiphase post-contrast MRI and MR angiogram revealed a large AVM draining into the right internal iliac vein and possibly into superior rectal vein. The main feeding artery was not visualised due to technical limitations of the study.

**Fig. 7:**
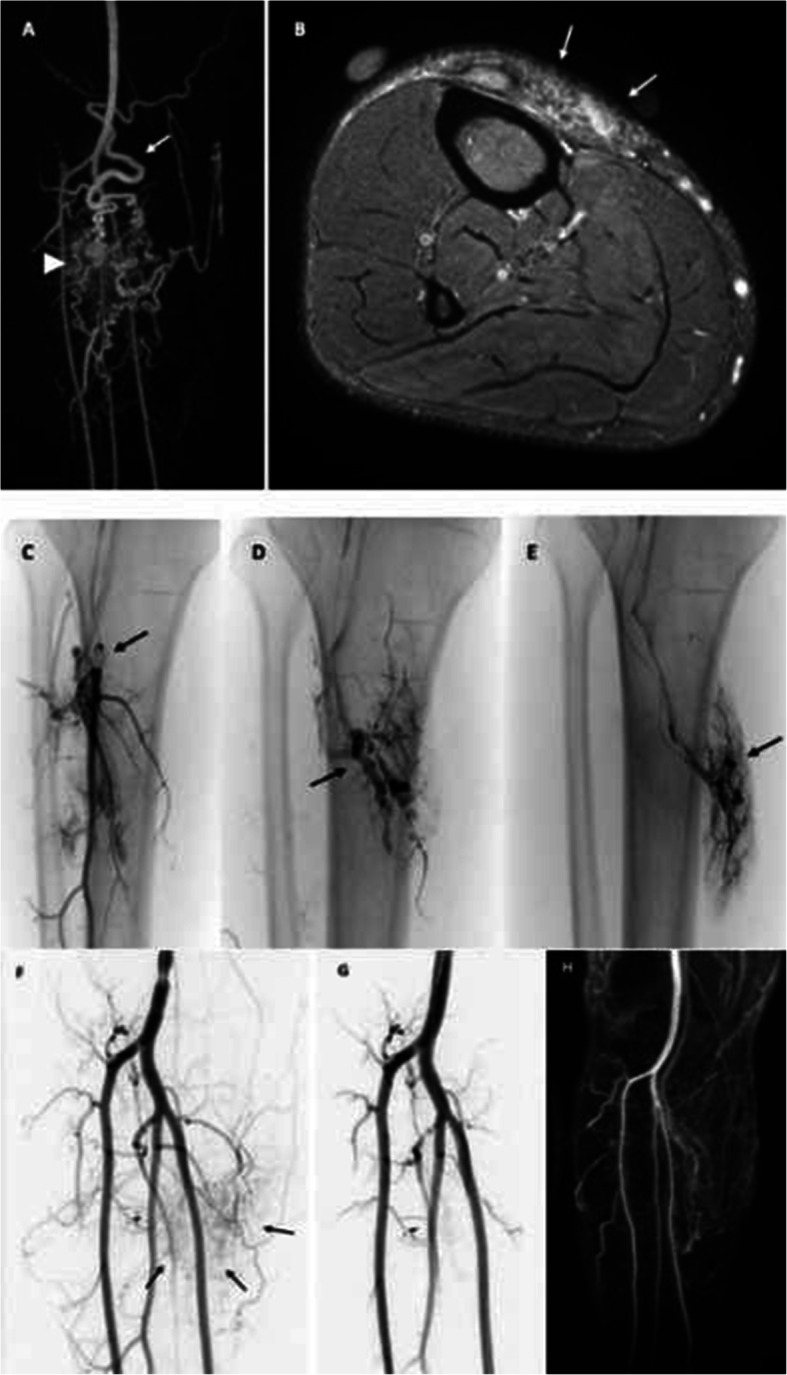
Patient 6 (uterine AVM). a) and b) Digital subtraction angiogram at the time of arterial injection demonstrates a large high flow nidus arising from the uterine artery. c) Arterial inflow occluded with the (Scepter XC, Microvention Inc.) occlusion balloon. d) angiography post initial treatment demonstrated complete nidal penetration treatment following slow infusion with Onyx

The procedure was performed under GA. Retrograde 8Fr access of the left CFA was achieved and a 6Fr intermediate catheter (Envoy DA, Codman Neuro) was placed into the right internal iliac artery. A 5Fr intermediate catheter (Sofia-Plus, Microvention Inc.) was subsequently placed into the right uterine artery ostium. A dual-lumen microballoon occlusion catheter (Scepter XC, Microvention) was then placed into the dominant arterial feeder of the high-flow uterine AVM and embolization was performed from the arterial side using a slow infusion of Onyx-18 with complete nidal penetration (balloon-assisted pressure cooker technique). Postprocedural angiogram demonstrated no other arterial feeder (including on the contralateral side) (Fig. 7). The patient was discharged the following day with no complications. At 3-month follow up the patient reported no further PV bleeding. Follow up ultrasound demonstrated reduction in the size of the AVM with normal doppler velocities.

### Case 7 – lower limb high-flow arteriovenous malformation

A patient presented with progressive swelling and pain over the right shin. Contrast-enhanced T2 fat saturated MR imaging demonstrated dilated and arterialised superficial draining veins over the proximal shin. Subsequently performed 4D time-resolved imaging of contrast kinetics (TRICKS) MRA showed a diffuse AVM in the proximal calf with a nidus and fistula components draining into arterialised draining veins.

**Fig. 8:**
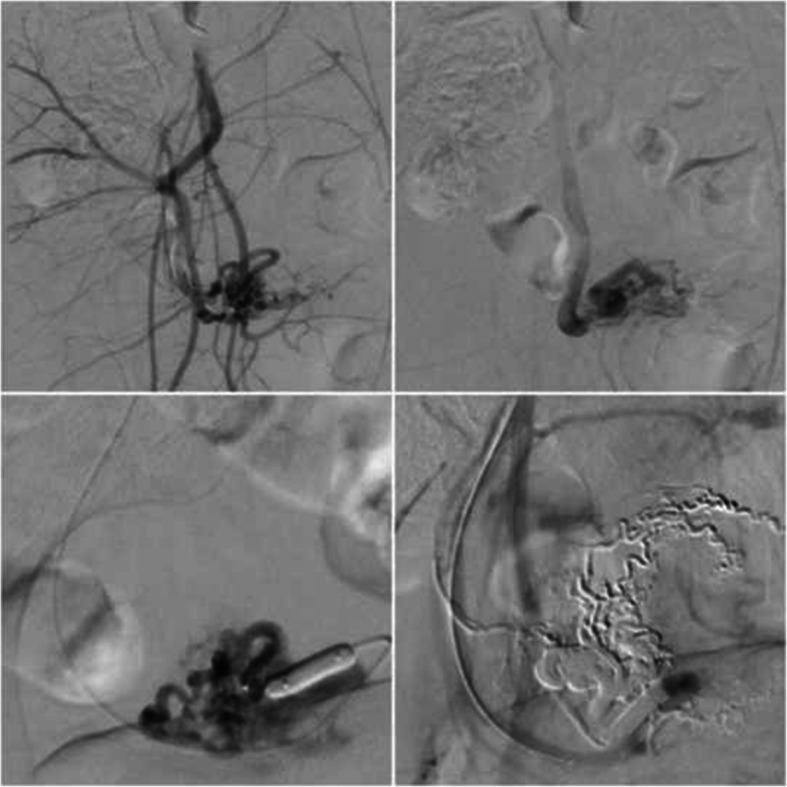
Patient 7 (Proximal calf AVM). (A) 4D time resolved imaging of contrast kinetics (TRICKS) MRA showing a diffuse AVM in the proximal calf with a nidus (arrowhead) and fistula components draining into arterialised draining veins (arrows). (B) Axial contrast-enhanced T2 fat saturated image showing dilated and arterialised superficial draining veins (arrows) over the proximal shin. Following antegrade right CFA access, DSA was performed with a balloon occlusion microcatheter inflated within arterial feeders from the (C) peroneal, (D) posterior tibial and (E) tibioperoneal trunk arteries. In each run, there was opacification of the AVM nidus and no evidence of contrast washout into the draining veins. Polidocanol 3% liquid embolic was injected under fluoroscopic control until nidal staining and stasis was observed. (F) Pre-embolisation DSA showing the nidal blush of the AVM and corresponding arterial feeders. (G) Post-embolisation DSA showing absence of any residual AVM nidus. (H) Follow-up MRA 8 weeks post-embolisation in the late arterial phase showing complete resolution of the AVM nidus and absence of early venous filling

Under GA, antegrade 6Fr access of the right CFA was achieved and a 5Fr diagnostic catheter was placed in the popliteal artery DSA demonstrated a large AVM with arterial feeders from the peroneal, posterior tibial and tibioperoneal trunk arteries. In each run, there was opacification of the AVM nidus and no evidence of contrast washout into the draining veins. A 2.8Fr microballoon occlusion catheter (Occlusafe, Terumo) was inflated within the arterial feeders and polidocanol 3% liquid embolic mixed with iodinated contrast was injected under fluoroscopic control until nidal staining and stasis was observed using a balloon-occlusion pressure cooker technique. No residual nidus was observed on postembolisation DSA. At 1 month postprocedural follow up, the patient reported considerable improvement in pain with persistent swelling which is expected to decrease over time. Follow-up MRA at 8 weeks confirmed complete obliteration of the treated AVM nidus (Fig. 8).

### Summary of results

Seven patients with symptomatic peripheral VMs were treated. This included five peripheral limb AVMs, one tongue AVM and one uterine AVM. Table 1 outlines the technical approach, complications and both clinical and imaging follow up. Intervention resulted in symptomatic improvement in all patients with immediate improved angiographic appearances and improved delayed MRI appearances.

**Table 1:** Patient demographics, AVM characteristics, technical approach, complications and clinical/imaging follow up

Patient	Type/Location	Symptoms	Approach	Fluoroscopy time (mins)	Embolic agent	Complications	Follow up time (months)	Symptomatic improvement (Y/N)	Clinical follow up outcome	Imaging follow up
1	Low-flow AVM, calf	Lower limb thrombus, pain	Arterial and percutaneous	23.7	Liquid sodium tetradecyl sulphate, 3%	Nil	3 months	Y	Improved exercise tolerance, discoloration and pain	No residual arterial component on MRI
2	High flow AVM, ankle	Pain	Arterial and venous	39.3	PHIL	Nil	6 months	Y	No ongoing pain or swelling, reduction in size, decreased discoloration	Significant reduction in size (only few small arterial feeders persisting) on MRI
3	High flow AVM, Elbow	Pain and swelling	Arterial and venous	70.7	Onyx and PHIL	Nil	2 years	Y	AVM recurrence requiring repeat endovascular treatment	Planned for 6 months postprocedure
4	Low flow venous malformation, tongue	Recurrent oral bleeding, aesthetically displeasing for patient	Arterial and percutaneous	15.7	Liquid sodium tetradecyl sulphate, 3%	Oropharyngeal swelling, tracheostomy	1 year	Y	Some residual symptoms including oral fullness and occasional ulceration and bleeding	Significant reduction in size of lingual compartment on MRI with residual arterial flow and patency in compartment at the floor of the mouth (smaller compared to preprocedural imaging)
5	High flow AVM, hand	Right arm pain, venous distension	Arterial and venous	114.7	Onyx and PHIL	Postprocedural haematoma, arterial dissection	9 months	Y	Improvement in pain, normal hand function	Some persistent arterialised flow extending from embolic material suggesting some AVM residium on postprocedural CT angiogram
6	High flow AVM, uterine	Large PV bleeding	Arterial and venous	273	Onyx	Nil	3 months	Y	No further irregular PV bleeding	N/A
7	High flow AVM, calf	Swelling and pain	Arterial	Not available	Polidocanol 3% liquid embolic	Nil	1 month	Y	Improvement in pain/ discomfort. Some ongoing swelling	Complete obliteration of AVM

Two complications occurred (cases 4 and 5). Case 4 had expected severe postprocedural swelling requiring ICU admission and tracheostomy. Whilst airway swelling was expected, the progression to tracheostomy was not. Fortunately the patient recovered and had improved imaging appearances. Case 5 required surgical evacuation of access-site haematoma and repair of arteriotomy and venotomy. This occurred in the context of very difficult venous access with multiple arterial punctures and the administration of thrombolysis. No long-term sequelae from these complications were seen in either of these cases. All patients had symptomatic improvement of their AVM and either complete or partial reduction in flow and shunting on repeat vascular imaging (excluding case 7 who had no follow up imaging).

## Discussion

Endovascular treatment of peripheral VMs is becoming increasingly adopted in tertiary centres for both high and low flow lesions. The use of LEAs allows for efficient and often complete obliteration of the nidus. The technical approach to peripheral VMs can be difficult due to complicated vascular anatomy including multiple arterial feeders and multiple venous outflow vessels including to the deep venous system.

Most VMs are congenital and display unpredictable growth patterns (Whitehead et al., 2013). Typical symptoms at presentation include pain, cosmetic issues or circulatory compromise (Vaidya et al., 2008). High-flow pressure effects of the VM may cause localised soft tissue destruction or erosion. Eventually systemic effects may cause cardiac failure (Igari et al., 2012). AVM treatment is usually governed by symptoms and patient preference (Mulliken & Glowacki, 1982a; Ernemann et al., 2010).

Cyanoacrylates are glues which polymerise when exposed to water or blood. This causes an acute inflammatory reaction with subsequent granulomatous change and fibrous reaction (Pollak & White, 2001). Cyanoacrylates have been used in AVM treatment. Traditional histoacryl glue is brittle whereas more novel glue agents are more stable and polymerise more slowly allowing for nidal penetration. Onyx is a copolymer of ethylene vinyl alcohol prepared with dimethyl sulfoxide (DMSO) as a solvent, with the addition of tantalum powder making it radiopaque (Vanninen & Manninen, 2007; Saeed Kilani et al., 2015). Precipitating hydrophobic injectable liquid (PHIL) is another biocompatible polymer dissolved in DSMO solvent and covalently bonded with iodine (Vollherbst et al., 2018b). Co-polymerisation and solidification of these agents occur when they comes into contact with ionic fluid such as blood. While both agents are largely non-adhesive and cohesive they can still embolise in high flow situations and rarely adhere to the catheter even if they reflux around the catheter tip. If reflux does occur, such as in high pressure situations, this can lead to issues of deflation and withdrawal. Detergent sclerosants such as sodium tetradecyl sulfate and polidocanol can also be used in the treatment of VMs as seen in this case series.

The original pressure cooker technique was first described by Chapot et al. and was designed to create an anti-reflux mechanism using a plug of coils and glue during liquid embolisation of brain AVMs (Chapot et al., 2014). The plug was placed between the tip and the detachment zone of a previously placed DMSO-compatible microcatheter using a second non-detachable microcatheter. In addition to reducing the risk of non-target embolisation, this technique provided better quality angiography and increased the ability to achieve deep nidal penetration.

A modified pressure cooker technique (mPCT) and transvenous pressure cooker technique (tPCT) have also been described (Zhang et al., 2017; Abud et al., 2016). In the mPCT, catheter placement is the same however EVOH in DSMO embolisation through the distal microcatheter is performed first and no coils are used. As Onyx begins to reflux, a glue plug is created between the column of onyx and the detachable mark of the distal microcatheter. The tPCT involves the deployment of coils into the draining vein prior to embolisation. Arterial inflow is then controlled using a balloon occlusion catheter followed up Onyx injection. Balloon-assisted pressure cooker techniques have been described for the treatment of VMs in simulated settings with promising results (Arslan, 2000; Gentric et al., 2015; Kim et al., 2017; Vollherbst et al., 2018a). These techniques have also been described in successfully treating central nervous system VMs, however limited data exists regarding their safety and efficacy for peripheral VMs.

Dual-lumen balloon microcatheters such as the Scepter C and XC (Microvention) are DMSO compatible and allow for occlusion of inflow vessels and can accommodate an 0.014″ guidewire. Commonly used 0.014 guidewires that are compatible with the Scepter platform include the Synchro Standard or Soft 0.014200 cm microwire (Stryker Neurovascular, MI, USA). These wires are composed of a central stainless-steel core with a platinum-tungsten alloy coil tip allowing for excellent trackability and fluoroscopic visualisation. The nitinol component improves torquability. These wires are commonly in neurointervention to access distal tortuous intracranial vessels making them ideal for selecting feeding arteries within a peripheral VM. The Scepter C and XC are available in 3 sizes and 1 size respectively. The Scepter XC also has some smaller specifications. Scepter C has a larger balloon inflation volume of 0.16–0.22 cc, whilst Scepter XC inflates to 0.10 cc. Balloon length also varies from 10 to 20 mm for the Scepter C, and is 11 mm for the Scepter XC. Both have a maximum burst pressure of 700 psi. The ability to inflate a balloon simultaneously during injection of embolic agents facilitates high-pressure filling of the AVM nidus and small arterial feeding vessels resulting in increased and more efficacious nidal obliteration. Another advantage of the Scepter microcatheter device is the ability to superselect arteries without the need to utilise a second device if balloon occlusion is needed to prevent reflux and non-target embolisation.

The Occlusafe balloon microcatheter (Terumo) is similar to the Scepter devices and can be used to achieve arterial occlusion in the peripheries. It is a 2.8Fr microcatheter ranging in length from 110 to 150 cm. It possesses a compliant polyurethane occlusion balloon on its tip measuring 10 mm in length with a maximal diameter of 4 mm. The Occlusafe balloon microcatheter requires a catheter with a minimum lumen size of 1.02 mm and thus can be used with standard 4 or 5Fr angiographic catheters with a 0.038″ lumen. Similarly to the Scepter devices, it is compatible with a 0.014″ guidewire. Venous occlusion can be achieved using compliant balloon-occlusion catheters such as the Copernic (BALT) or embolectomy balloon catheters. Dedicated occlusion balloons allow for smooth device delivery and the super-compliant nature of the balloon minimises the risk of vascular injury.

Despite these advantages, there are still limitations to this device. The balloon occupies a portion of the vascular lumen, which becomes a working dead space. Although reflux of embolic agent is prevented, on occasion the balloon may be difficult to remove. Traction may be applied at risk of vascular rupture and haemorrhage and in the case of catheter entrapment the microcatheter may be cut near the point of access and left in the vascular system. Catheter fracture during removal puts the patient at risk of thromboembolic complications and arterial occlusion. Retrograde filling of other arterial branches and thus non-target embolization is also possible.

One previously published case series (*n* = 4) has examined the use of the Scepter C device with Onyx embolisation for the treatment of peripheral vascular lesions, describing similar results (Jagadeesan et al., 2014). Endovascular treatment resulted in clinical and radiological improvement in three cases with follow up pending for the remaining patient. Two patients in this series required repeat embolisation, with the same technique employed. Non-target embolisation occurred in one patient resulting in digital infarction.

We report 7 cases with near complete symptomatic resolution in all cases. Our series employed a varied procedural approach with both percutaneous and intravascular embolic delivery. Variations to this could involve pressure cooker with venous control and arterial or venous injection (endoluminal or percutaneous), percutaneous injection with or without arteriovenous control of the flow and endoluminal venous injection only. Our modified ‘pressure-cooker’ technique could be used to treat any sort of AVM apart from those that are angiographically occult (such as capillary haemangiomas), particularly those that are high-flow and those with fistulous components.

Pure venous lakes could also be treated this way as seen in cases 1 and 4. Our decision to use this technique in these cases was to achieve a thorough angiographic assessment followed by precise arterial and venous control during embolisation/injection. Our view is that a simple percutaneous direct-stick approach is risky as the exact angiographic architecture is often not fully demonstrated on preprocedural imaging, especially if real-time sequences such as postcontrast TRICKS are not routine. The outflow control also avoids or minimises tourniquet use and increases stagnation time for sclerosant injection, avoiding counterproductive washout. In our experience we have seen very small arterial contributors to ‘low flow’ malformations on DSA which were not appreciable on postprocedural imaging. Direct-stick treatment of these lesions may lead to arterial or capillary obliteration with subsequent tissue ischaemia. There is also the risk of ‘pressurising’ a low flow malformation with retrograde leak of embolic material into the arteries. A further advantage is that it allows for repeat imaging if there is movement or concern for non-target embolisation.

One major complication occurred in a technically difficult patient in which surgical intervention was required. Given the anticipated airway compromise in case 4 despite aggressive corticosteroid therapy, ICU admission was expected. Future treatments in this patient may require a staged approach across several sessions to mitigate further risk of airway compromise with a single staged approach. Additionally, choice of embolic agent in this situation may be important. One of our cases required re-treatment due to symptomatic AVM recurrence reinforcing the need for continued clinical and imaging follow-up of treated lesions.

Our series is limited by a small sample size, however our results suggest technical feasibility and safety with promising preliminary results across a variety of high and low-flow VMs. Larger studies with longer follow-up would aid in assessing long-term efficacy and allow comparison of results with those of conventional techniques.

## Conclusion

Neuroendovascular techniques using microballoon occlusion catheters can be safely employed in the treatment of peripheral VMs, resulting in technical and clinical success. The implementation of these techniques for the treatment of peripheral VMs may reduce the need for repeat embolisation.

## Data Availability

Data not shared (hospital database)
